# Disease-modifying effects of phosphocitrate and phosphocitrate-β-ethyl ester on partial meniscectomy-induced osteoarthritis

**DOI:** 10.1186/s12891-015-0724-x

**Published:** 2015-09-30

**Authors:** Yubo Sun, Nikkole Haines, Andrea Roberts, Michael Ruffolo, David R. Mauerhan, Kim L. Mihalko, Jane Ingram, Michael Cox, Edward N. Hanley

**Affiliations:** Department of Orthopedic Surgery, Carolinas Medical Center, PO Box 32861, Charlotte, NC 28232 USA; Department of Comparative Medicine, Carolinas Medical Center, PO Box 32861, Charlotte, NC 28232 USA

**Keywords:** Calcification, Crystals, Osteoarthritis, Phosphocitrate, MMP-13, ADAMTS5, CCL-5

## Abstract

**Background:**

It is believed that phosphocitrate (PC) exerts its disease-modifying effects on osteoarthritis (OA) by inhibiting the formation of crystals. However, recent findings suggest that PC exerts its disease-modifying effect, at least in part, through a crystal-independent action. This study sought to examine the disease-modifying effects of PC and its analogue PC-β-ethyl ester (PC-E) on partial meniscectomy-induced OA and the structure-activity relationship.

**Methods:**

Calcification- and proliferation-inhibitory activities were examined in OA fibroblast-like synoviocytes (FLSs) culture. Disease-modifying effects were examined using Hartley guinea pigs undergoing partial meniscectomy. Cartilage degeneration was examined with Indian ink, safranin-O, and picrosirius red. Levels of matrix metalloproteinase-13 (MMP-13), ADAM metallopeptidase with thrombospondin type 1 motif 5 (ADAMTS5), chemokine (C-C motif) ligand 5 (CCL5), and cyclooxygenase-2 (Cox-2) were examined with immunostaining. The effects of PC-E and PC on gene expressions in OA FLSs were examined with microarray. Results are expressed as mean ± standard deviation and analyzed using Student’s *t* test or Wilcoxon rank sum test.

**Results:**

PC-E was slightly less powerful than PC as a calcification inhibitor but as powerful as PC in the inhibition of OA FLSs proliferation. PC significantly inhibited cartilage degeneration in the partial meniscectomied right knee. PC-E was less powerful than PC as a disease-modifying drug, especially in the inhibition of cartilage degeneration in the non-operated left knee. PC significantly reduced the levels of ADAMTS5, MMP-13 and CCL5, whereas PC-E reduced the levels of ADAMTS5 and CCL5. Microarray analyses revealed that PC-E failed to downregulate the expression of many PC-downregulated genes classified in angiogenesis and inflammatory response.

**Conclusions:**

PC is a disease-modifying drug for posttraumatic OA therapy. PC exerts its disease-modifying effect through two independent actions: inhibiting pathological calcification and modulating the expression of many genes implicated in OA. The β-carboxyl group of PC plays an important role in the inhibition of cartilage degeneration, little role in the inhibition of FLSs proliferation, and a moderate role in the inhibition of FLSs-mediated calcification.

## Background

Osteoarthritis (OA) is a heterogeneous and multifactorial degenerative joint disease characterized by gradual loss of articular cartilage, formation of osteophytes, and synovial inflammation. Current non-surgical treatments for OA, such as non-steroid anti-inflammatory drugs and steroid injections, only relieves pain, inflammation, and effusion. There is a need for the development of disease-modifying drugs that can not only relieve pain and inflammation, but also inhibit cartilage degeneration. The lack of progress in the development of disease-modifying drugs is largely due to our limited understanding of the pathogenesis of OA and insufficient knowledge regarding the molecular targets for therapeutic intervention.

The biochemical events involved in OA are poorly understood. Many extracellular matrix degrading enzymes and inflammatory cytokines, including matrix metollproteinase-13 (MMP-13), ADAM metallopeptidase with thrombospondin type 1 motif 5 (ADAMTS5), interleukin-1 (IL-1), and cyclooxygenase-2 (Cox-2) have been implicated in OA [1–3]. Pathological calcification has also been implicated. Basic calcium phosphate crystals and calcium pyrophosphate dihydrate crystals are the two most common articular calcium-containing crystals. The presence of these crystals within the knee joints of end-stage OA patients is well recognized [4–9]. Injection of these crystals into the knee joints of dogs and mice induce a severe inflammatory response [10, 11]. These crystals also induced cell mitogenesis and stimulated the production of matrix metalloproteinases (MMPs), nitric oxide, and inflammatory cytokines [12–15], suggesting that crystals may play a role in the development or progression of OA.

Phosphocitrate (PC) is a naturally occurring small molecule originally identified in rat liver mitochondrial extract [16]. Since its original identification, PC has been shown to be a powerful calcification inhibitor [17, 18]. PC prevented soft tissue calcification and didn’t produce any significant toxic side effect in rats in doses up to 150 μmol/kg/day [19]. In addition, PC inhibited crystal-induced mitogenesis, expression of MMPs, and cell death [20–22]. Based on these findings, a hypothesis that PC is a disease-modifying drug for calcification-induced OA therapy was postulated [23]. A subsequent study demonstrated that PC inhibited meniscal calcification, and that a decrease in meniscal calcification was accompanied with reduced cartilage degeneration in Hartley guinea pigs (calcification-induced OA), but had no significant effect on cartilage degeneration in partial meniscectomy-induced OA in rabbit (posttraumatic OA or non-calcification induced OA) [24]. The investigators concluded that PC is a disease-modifying drug for calcification-induced OA therapy but not for non-calcification-induced OA. It was believed that PC exerted its OA disease-modifying activity by inhibiting the formation of articular calcium crystals and the detrimental interaction between these crystals and joint cells (crystal-dependent action) [23, 24]. Although this theory was well received at the time, doubt emerged upon the findings that bisphosphonates, which were potent calcification inhibitors [25, 26], failed to inhibit cartilage degeneration in animal models of OA, including Hartley guinea pig model of calcification-induced OA [27, 28].

We recently found that PC downregulated the expression of many genes classified in cell proliferation, angiogenesis, and inflammatory response, while upregulating the expressions of many genes classified in skeletal system development in the absence of calcium crystals [29–31]. These newer findings suggest that crystal-dependent action of PC may not be a sole action underlying the OA disease-modifying effect of PC. It is likely that PC exerts its OA disease-modifying activity through two independent actions: i) inhibiting the formation of crystals and crystal-induced expressions of MMPs (a crystal-dependent action), and ii) modulating the expressions of genes implicated in OA (a crystal-independent action).

**Fig. 1:**
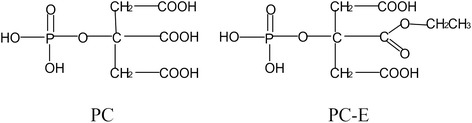
Molecular structures of PC and PC-E

PC-β-ethyl ester (PC-E) is a PC analogue where a β-carboxyl group is replaced by an ethyl ester group (Fig. 1). We are interested in PC-E not only because compared to PC, PC-E has less negative charges, therefore it may be more easily absorbed in the intestine if administered through the oral route, but also because novel new PC analogues may be prepared by linking other active group(s) to this carboxyl group. In this study, we sought to examine disease-modifying activity of PC and PC-E on posttraumatic OA and investigate the structure-activity relationship. The results of this study may not only provide information valuable for the design and development of new PC analogues as disease-modifying drug for OA therapy, but also for a better understanding of pathogenesis of OA and the molecular mechanism underlying the disease-modifying activity of PC.

## Methods

Dulbecco’s minimum essential medium (DMEM), fetal bovine serum, stock antibiotic/antimycotic mixture were obtained from Invitrogen (Carlsbad, CA). ^45^Calcium was obtained from Perkin-Elmer (Boston, MA). Antibody specific to MMP-13 (Lifespan Biosciences, Seattle, WA), ADAMTS5 (Santa Cruz Biotechnology, Dallas, TX), Cox-2 (Santa Cruz Biotechnology, Dallas, TX), and CCL-5 (Bioss, Woburn, MA) were obtained from the commercial sources indicated. Safranin-O, fast green, picrosirius red, and alcian blue were obtained from Polysciences (Warrington, PA). PC and PC-E were prepared according to the method described [32]. All other chemicals were obtained from Sigma-Aldrich (St. Louis, MO).

### Calcification assay

OA fibroblast-like synoviocytes (FLSs), similar to OA chondrocytes, play a role in the formation of articular crystal [9, 33, 34]. To compare the calcification-inhibitory activity of PC and PC-E, we performed an ATP-induced calcification assay using telomerase immortalized human OA fibroblast-like synoviocytes (FLSs), hTERT-OA 13A FLSs [9]. Briefly, hTERT-OA 13A FLSs were plated in a 24 well cluster plate at 95 % confluence. On the second day, DMEM with 10 % serum was changed to DMEM containing 0.5 % serum. On the third day, after cells became quiescent, DMEM containing 1 mM ATP and trace-labeled with 1 μCi/ml ^45^calcium was added. Immediately, increasing amounts of PC, PC-E, or citrate were added into the wells. Forty-eight hours later, cells were washed with cold Hank’s balanced salt solution five times and lysed with 0.1 N NaOH. Radioactivity of lysate in each well was quantified using liquid scintigraphy. Calcification-inhibitory activity of disodium ethane-1-hydroxy-1, 1-diphosphonate (EHDP) was also examined. Results were presented as the mean ± SD of five independent experiments.

### Proliferation assay

Proliferation-inhibitory activities of PC-E and PC were examined as described [29]. Briefly, hTERT-OA 13A FLSs (4×10^4^) were plated in six well cluster plates. On the second day, DMEM containing 10 % serum and PC-E (0.5 mM) or PC (0.5 mM) was added into the top three wells. DMEM containing 10 % serum without PC-E or PC was added into the bottom three wells as a control. DMEM was changed every three days until the cells in the bottom wells reached 85 % confluence (cultured for about 12 to 14 days). All cells were then harvested and the cell number in each well was counted. Results were presented as the mean ± SD of three independent experiments.

### Experimental animals

This study was performed according to the guidelines set forth by the Institutional Animal Care & Use Committee of Carolinas Medical Center, which approved the animal protocol. Male Hartley guinea pigs at three weeks of age were obtained from Charles River Laboratories (Wilmington, MA) and individually housed in 29 × 21 × 10 inch solid bottom cages. Guinea Pig Chow (No. 5025; Ralston Purina, Richmond, Indiana) and water were available *ad libitum*. The first group of guinea pigs (*n* = 5) received intraperitoneal injection of PC (40 mg/kg) twice per week, second group (*n* = 5) received PC-E (40 mg/kg) and the last group (*n* = 5) received physiological saline. Two months later, partial medial meniscectomy was performed on the right knee of all guinea pigs to induce posttraumatic OA. A week after the surgery, injection of PC, PC-E, or saline was resumed. Five months later, these guinea pigs were euthanized by the administration of Euthasol (Virbac Animal Health, Ft. Worth, Texas). Hind limbs were collected, fixed in 10 % formalin, and transferred to 70 % ethanol until use. Structural changes in the articular cartilage of Hartley guinea pigs were not observed until three months of age [35], therefore pretreatment of the young guinea pigs with PC or PC-E for two months before partial meniscectomy surgery will not result in detectable structural changes in the articular cartilage.

### Radiographic, microscopic, and histological examinations

Radiographs of knee joints were obtained with a digital radiography system (piXaray 100, Bioptics Inc., Tucson, AZ). After dissection of the knee joints, radiographs of medial meniscus were obtained. All tibia plateaus were first stained with Indian ink as described [36]. The tibia plateaus were then decalcified in Cal-Ex II solution (Fisher Scientific, Fairlawn, NJ) and cut coronally in the center to produce two equal portions. The posterior portion was embedded in paraffin and sectioned with a Leica RM2025 microtome (Nussloch, Germany) to obtain 4 μm sections. Three non-consecutive sets of sections (three consecutive sections in each set) obtained at 400 μm intervals were stained with safranin-O and counter stained with fast green. These sections (nine sections for each cartilage specimen) were graded according to standard Mankin criteria with minor modifications [37]. Two sections in each cartilage specimens were also stained with picrosirius red and counter stained with alcian blue.

### Cartilage thickness

Central portion of safranin-stained sections (the most degenerative area) was photographed and the area of cartilage (same length for each section) was measured using the measuring tool in Adobe acrobat software (San Jose, CA). Briefly, the image file was opened with Adobe acrobat and the cartilage was traced continuously with the “pen” in the measuring tool along the irregular surface of the articular cartilage (numerous very short straight lines were connected together to form an irregular line) and the border between cartilage and subchondral bone. After tracing the cartilage was finished, the area was automatically calculated. Cartilage thickness is obtained by dividing the area with the length of the cartilage measured.

### Immunohistochemistry

Two sections in each tibia plateau were deparaffinized with xylene and rehydrated with graded ethanol. Endogenous peroxidase activity was blocked by incubation with deionized water containing 3 % H_2_O_2_ for five minutes. Non-specific binding was blocked by incubation with 100 μl of 10 % normal horse serum diluted in base solution (4 % BSA and 5 % non-fat dry milk in PBS) for 20 min. These sections were incubated with primary antibodies (1:100 dilution) for one hour, followed with secondary reagent for 30 min (Immpress reagent kit, Vector, Inc., Burlingame, CA). Negative control was performed using mouse IgG. Slides were rinsed in phosphate buffered saline three times and visualized with 3, 3′-diaminobenzidin. Slides were counterstained with light green, dehydrated and mounted with resinous mounting media. These slides were graded on a scale of 0–4, where 0 = very weak staining; 1 = weak staining; 2 = moderate staining; 3 = strong staining; 4 = very strong staining as described [38].

### Microarray

Briefly, hTERT-OA 13A FLSs [9] were plated in two 100 mm plates at 90 % confluence. On the second day, medium was changed to medium containing 1 % serum. On the third day, medium containing 1 % serum and PC-E (0.6 mM) was added to a plate, and medium containing 1 % serum but without PC-E was added to the other plate. Twenty-four hour later, total RNA was extracted using Trizol reagent (Invitrogen, Carlsbad, CA) and purified using Oligotex kit (Qiagen, Valencia, CA). These RNA samples were used for microarray as described [29]. Microarray analysis of PC on gene expressions has been performed previously [29].

### Statistical analyses

Results of calcification assay, proliferation assay, and cartilage thickness measurements (variables measured on continuous interval) are presented as the mean ± SD. The differences between the results in 2 groups were analyzed using Student’s *t* Test. Scores of histological staining and immunostaining (variables presented as ordinal data) were presented as the mean ± SD. The differences between the scores in two groups were analyzed using Wilcoxon rank sum test. Statistical analysis was performed using the statistical analysis tool in the Sigma Plot software, version 12 (Systat Software, Inc., San Jose, CA).

## Results

### Calcification- and proliferation-inhibitory activities

**Fig. 2:**
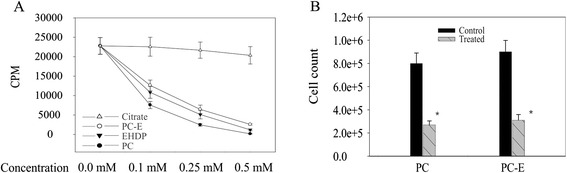
Calcification- and proliferation-inhibitory activities. **a** PC, PC-E, and EHDP inhibit OA FLSs-mediated calcification. CPM (count per minute) in control, PC-E, PC and EHDP treated cells are 22,784 (±2,156), 6,452 (±1,095), 2,466 (±352), and 5,124 (±1114), respectively, at the concentration of 0.25 mM. **b** PC and PC-E inhibit OA FLSs proliferation. Cell numbers in control and PC treated wells are 845,236 (±89,521) and 285,706 (±34,132). Cell numbers in control and PC-E treated wells are 924,484 (±98,718) and 331,096 (±48,694). *= *p* < 0.01, versus control

PC, PC-E, and EHDP, but not citrate, inhibited ATP-induced calcium deposition in a dose dependent manner (Fig. 2a). PC is the most potent calcification inhibitor, with PC-E being 19 % and EHDP being 13 % less powerful than PC in the inhibition of OA FLSs-mediated calcium deposition at the concentration of 0.25 mM (*p* < 0.01). PC-E, similar to PC [29], also inhibited the proliferation of human OA FLSs (Fig. 2b). There were about 66 % and 64 % fewer human OA FLSs in the PC and PC-E treated wells, respectively, compared to controls (*p* < 0.01). PC and PC-E had no effect on cell viability up to the concentration of 10 mM whereas EHDP caused cell death when its concentration was higher than 1.5 mM (not shown).

### Radiographic examinations

**Fig. 3:**
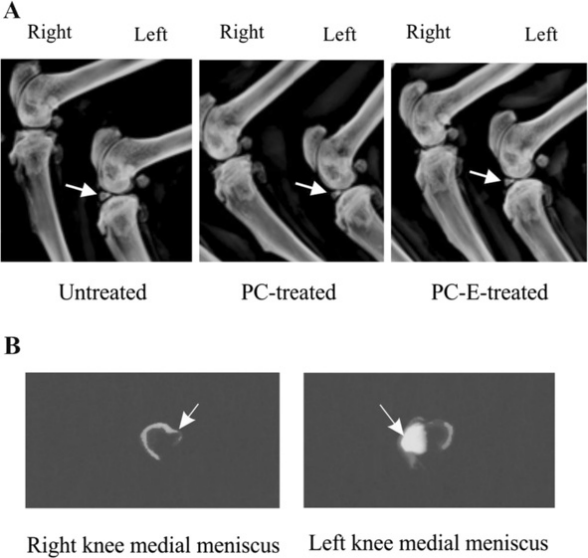
Radiographs of knees and medial menisci. **a** Representative radiographs of meniscectomied right knee and non-operated left knee of untreated, PC-treated, and PC-E treated guinea pigs. Calcified medial menisci are indicated by arrows. **b** Radiographs of the meniscectomied right knee medial meniscus and the non-operated left knee medial meniscus of an untreated guinea pig. Severely calcified anterior horn of the medial meniscus (arrow) is present in the left knee, but not in the right knee

Representative radiographs of the knee joints and medial menisci were provided in Fig. 3. As expected, calcified medial meniscus was observed in the non-operated left knee, but not in the meniscectomied right knee (Fig. 3a). Meniscal calcification predominantly occurred in the anterior horn of the medial meniscus (Fig. 3b). The severely calcified anterior horn of the medial meniscus was absent in the meniscectomied right knee. As shown in Fig. 3a, meniscus in the non-operated left knee of PC and PC-E treated guinea pigs were slightly less calcified compared to untreated guinea pigs, indicating that PC and PC-E reduced meniscal calcification. However, meniscal calcification in PC and PC-E treated guinea pigs appeared similar. No signs of cartilage calcification were observed.

### Microscopic examinations

**Fig. 4:**
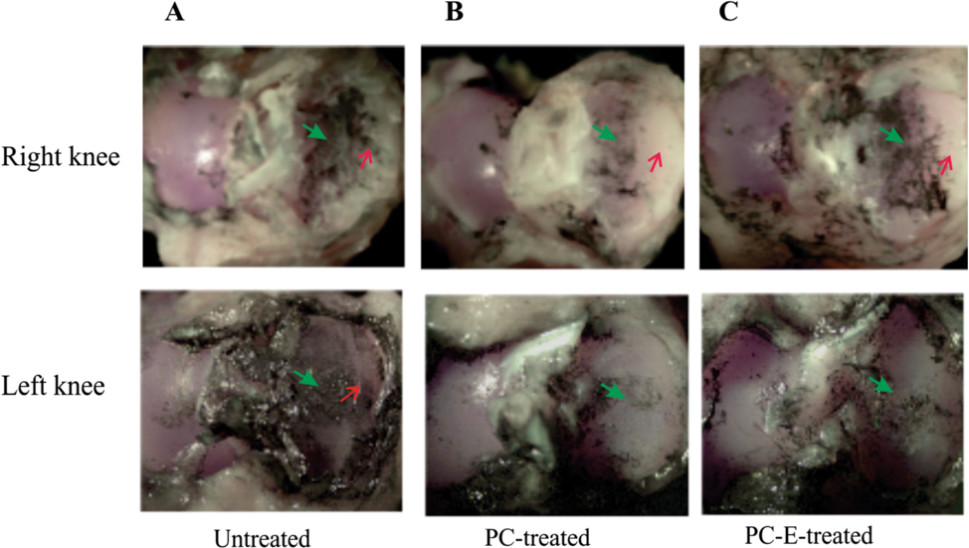
Representative images of Indian ink staining. **a** Indian ink-stained tibia plateaus of the meniscectomied right knee and the non-operated left knee of an untreated guinea pig. **b** Indian ink-stained tibia plateaus of the meniscectomied right knee and the non-operated left knee of a PC-treated guinea pig. **c** Indian ink-stained tibia plateaus of the meniscectomied right knee and the non-operated left knee of a PC-E-treated guinea pig. Green arrow - central area of medial tibia plateau. Red arrow - peripheral area of medial tibia plateau

Indian ink staining was used to examine the tibia plateaus of all guinea pigs. Representative Indian ink stained tibia plateaus are provided in Fig. 4. As shown, cartilage damage, visualized with the help of Indian ink, spanned a larger area in the medial tibia plateaus of the meniscectomied right knee than the cartilage damage in the medial tibia plateaus of the non-operated left knee (Fig. 4a). In the non-operated left knee, cartilage damage was confined in the central area of the medial tibia plateau (Green arrow) whereas in the meniscectomied right knee, cartilage damage was not only present in the central area (Green arrow) but also present in the peripheral area that was originally covered with meniscus (Red arrow). These results demonstrated that meniscal injury and joint instability resulted in increased cartilage degeneration, especially cartilage degeneration in the peripheral area, which was not observed in the non-operated left knee. As shown in Fig. 4b and c, there was less cartilage damage in the medial tibia plateau of the meniscectomied right knee, especially in the peripheral area, in PC and PC-E treated guinea pigs compared to untreated controls, indicating that PC and PC-E inhibited cartilage degeneration induced by meniscal injury and joint instability. There was also less cartilage damage in the medial tibia plateau of the non-operated left knee in PC and PC-E treated guinea pigs compared to untreated controls.

### Histological examinations

**Fig. 5:**
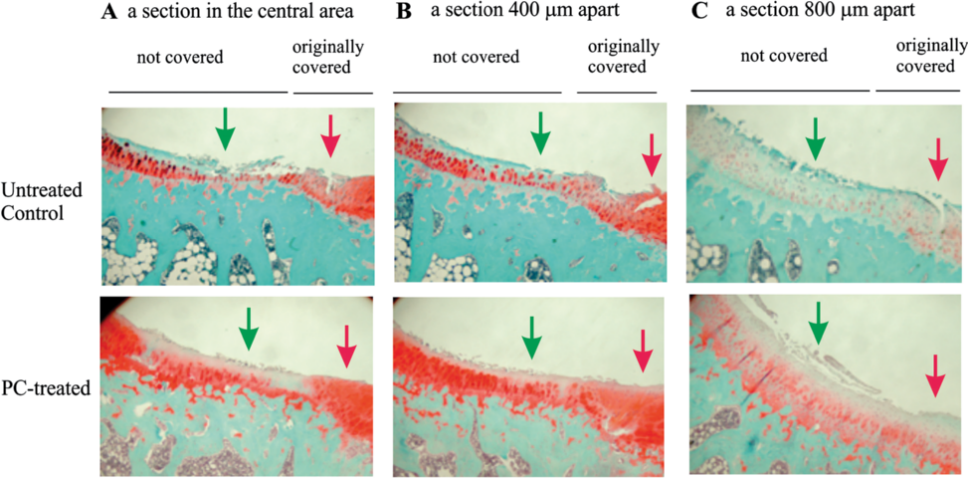
Safranin-O stained sections of medial tibia plateaus of meniscectomied right knee. **a** A section in the central area of medial tibia plateau of an untreated and a PC treated guinea pig. **b** Second section, 400 μm away from first section. **c** Third section, 800 μm away from first section. Green arrows - central area of medial tibia plateau. Red arrows - peripheral area

Representative three non-consecutive safranin-O stained sections from the meniscectomied right knee in untreated and PC treated guinea pigs were provided in Fig. 5. Consistent with Indian ink staining, safranin-O staining revealed that severe cartilage damage occurred in both the central and peripheral areas of medial tibia plateau in untreated guinea pigs. Cartilage damage and proteoglycan loss, in many cases, extended into deep and calcified zones. Cartilage damage and proteoglycan loss were significantly reduced in PC treated guinea pigs. In the central area of medial tibia plateaus, mild to moderate cartilage damage and proteoglycan loss were observed, whereas in the peripheral area, only mild cartilage damage and proteoglycan loss were observed. Consistent with Indian ink staining, cartilage damage and proteoglycan loss were also reduced in PC-E treated guinea pigs (photos not shown).

**Fig. 6:**
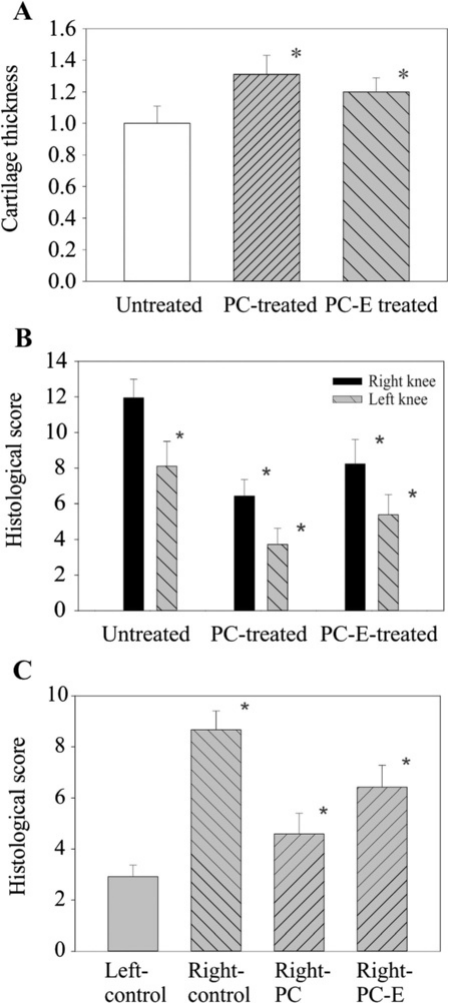
Cartilage thickness and histological scores of medial tibia plateaus. **a** Normalized cartilage thickness in untreated, PC-treated, and PC-E treated guinea pigs are 1 (±0.11), 1.31 (±0.13), and 1.18 (±0.10).* *p* < 0.01, versus controls. **b** Histological scores of medial tibia plateau in the meniscectomied right knee in untreated, PC, and PC-E treated guinea pigs are 11.95 (±1.05), 6.43 (±0.93), and 8.24 (±0.1.37). Histological scores of medial tibia plateau in the non-operated left knee in untreated, PC, and PC-E treated guinea pigs are 8.11 (±1.39), 3.72 (±0.91), and 5.74 (±0.12). * *p* < 0.05, versus controls. **c** Histological scores of the peripheral area of medial tibia plateau in the non-operated left knee and the meniscectomied right knee of untreated guinea pigs are 2.92 (±0.45) and 8.67 (±0.74). Histological scores of the peripheral area of medial tibia plateau in the meniscectomied right knee of PC and PC-E treated guinea pigs are 4.59 (±0.81) and 6.42 (±0.0.86). * *p* < 0.05, versus controls

In addition, cartilage in untreated guinea pigs appeared thinner than the cartilage in PC treated guinea pigs (Fig. 5). We measured the cartilage thickness of all guinea pigs as described. As shown in Fig. 6a, cartilage in PC treated guinea pigs was 31 % thicker than the cartilage in untreated guinea pigs (*p* = 0.01). Similarly, PC-E also inhibited cartilage thinning. Cartilage in PC-E treated guinea pigs was 18 % thicker than the cartilage in untreated guinea pigs (*p* = 0.02).

These safranin-O stained sections (nine sections for each tibia plateau specimen) were graded. As shown in Fig. 6b, PC and PC-E significantly reduced the histologic score of medial tibia plateau (black bars), resulting in a 46  and 30 % reduction in the histological scores, respectively (*p* < 0.05). For comparison, safranin-O stained sections in the non-operated left knee medial tibia plateau were graded. The histological score of the medial tibia plateau in the non-operated left knee was much lower than the histological score of the medial tibia plateau in the meniscectomied right knee (*p* < 0.01), confirming that meniscal injury and joint instability resulted in significantly increased cartilage damage. PC and PC-E reduced the histologic score of the medial tibia plateau in the non-operated left knee (grey bars), resulting in a 54  and 28 % reduction in the histological scores, respectively (*p* < 0.05).

Due to severe cartilage damage being observed in the peripheral area of the medial tibia plateau in the meniscectomied right knee but not in the non-operated left knee, we decided to grade the peripheral area specifically. As shown in Fig. 6c, meniscal injury and joint instability in the meniscectomied right knee resulted in severe cartilage degeneration in the peripheral area compared to the non-operated left knee. Cartilage damage in the peripheral area of the medial tibia plateau in the meniscectomied right knee was significantly reduced in PC and PC-E treated guinea pigs, indicating again that PC and its analogue inhibits injury and joint instability-induced cartilage degeneration.

**Fig. 7:**
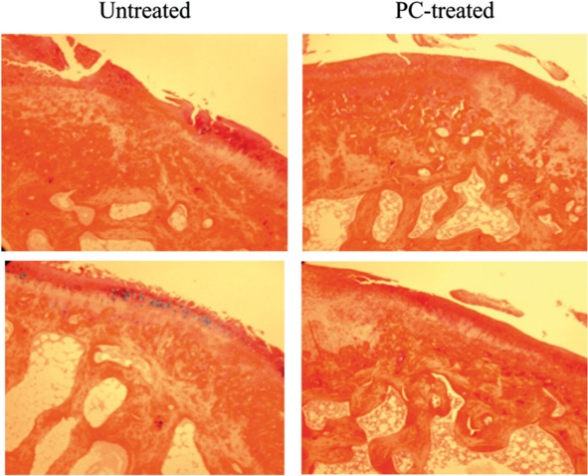
Picrosirius red stained sections. Left: section at the central area of the medial tibia plateau from two untreated guinea pigs. Right: section at the central area of the medial tibia plateau from two PC treated guinea pigs

Two sections in each tibia plateau were also stained with picrosirius red and counter-stained with alcian blue. Representative images of picrosirius red stained sections are provided in Fig. 7. As shown, severe cartilage damage was observed in the untreated guinea pigs and in many cases, cartilage lesions extended into the middle, deep, and calcified zones. In contrast, cartilage damage was much less prominent in PC treated guinea pigs. Surprisingly, unlike the severe loss of safranin-O staining, there was no severe loss of picrosirius red staining in untreated guinea pigs compared to PC treated guinea pigs. These findings indicate that OA cartilage in the untreated Hartley guinea pigs was characterized by breakdown of collagen fibers, not by collagen loss. Interestingly, alcian blue staining was observed in some chondrocytes residing in the middle zone of articular cartilage in the untreated guinea pig, which was not observed in the cartilage of PC-treated guinea pigs. One explanation for this finding is that some chondrocytes in OA cartilage synthesize more proteoglycans in response to matrix degeneration. Consistent with this explanation, the intensity of safranin-O staining in the nucleus and cytoplasm of some chondrocytes in the untreated guinea pigs appeared much higher than the intensity of safranin-O staining in the nucleus and cytoplasm of most chondrocytes in the PC-treated guinea pigs (Fig. 5). Severe loss of safranin-O staining in the articular cartilage of untreated guinea pigs mainly occurred in the extracellular matrix space (Fig. 5).

### Immunohistochemistry

**Fig. 8:**
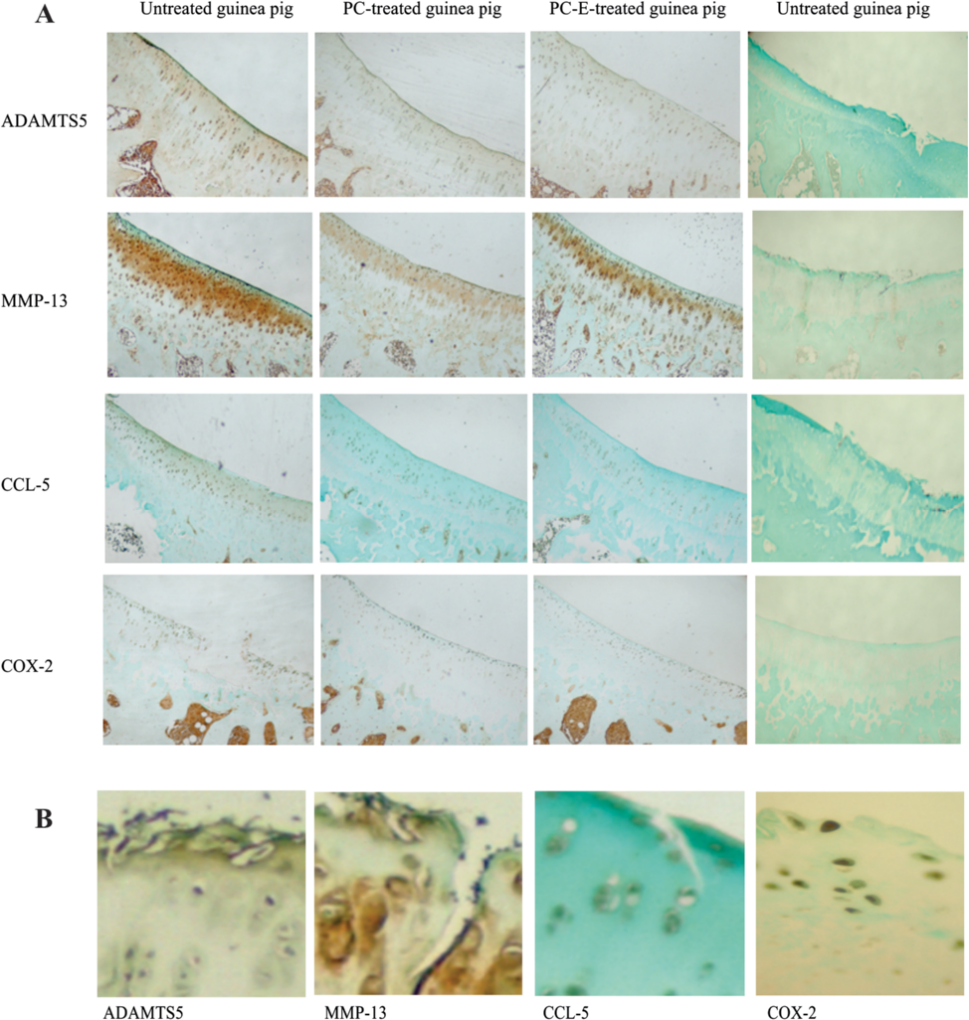
Immunostaining. **a** Immunostaining of ADAMTS5, MMP-13, CCL-5, and Cox-2. No staining was noted in negative control. **b** Enlarged images of immunostaining of ADAMTS5, MMP-13, CCL-5, and Cox-2 in untreated guinea pigs. ADAMTS staining was mainly found in the pericellular area and extracellular matrix, whereas MMP-13 staining was found in the nucleus, cytoplasm, pericellular area, and extracellular matrix. CCL-5 staining was found in the nucleus, cytoplasm, and pericellular area. Cox-2 staining was mainly found in the nucleus and cytoplasm

Representative images of immunostaining are provided in Fig. 8a. In untreated guinea pigs, high levels of ADAMTS-5 protein was present in superficial, middle, and deep zones, whereas high level of MMP-13 protein was present in the middle and deep zones. The level of ADAMTS5 protein was reduced in PC and PC-E treated guinea pigs. The level of MMP-13 protein was also reduced, especially in the middle zone in PC treated guinea pigs. However, the level of MMP-13 protein appeared only slightly reduced in PC-E treated guinea pigs. In untreated guinea pigs, CCL-5 protein was present in superficial, middle, and deep zones, whereas Cox-2 protein was present in superficial and middle zones. It was clear that the level of CCL-5 protein was reduced in PC and PC-E treated guinea pigs. However, the level of Cox-2 protein appeared only slightly reduced in PC and PC-E treated guinea pigs compared to the untreated controls.

**Fig. 9:**
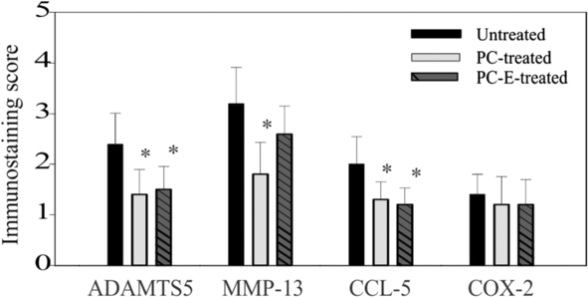
Scores of immunostaining. Scores for ADAMTS5 immunostaining in untreated, PC, and PC-E treated guinea pigs are 2.40 (±0.61), 1.40 (±0.49), and 1.50 (±0.45); Scores for MMP-13 immunostaining are 3.20 (±0.71), 1.80 (±0.64), and 2.70 (±0.55); Scores for CCL-5 immunostaining are 2.00 (±0.55), 1.30 (±0.35), and 1.20 (±0.33); Scores for Cox-2 immunostaining are 1.40 (±0.40), 1.20 (±0.55), and 1.20 (±0.49). *p < 0.05, versus controls

The scores of these immunostainings are provided in Fig. 9. As shown, PC and PC-E treatments resulted in 42 % and 38 % reductions in the immunostaining score of ADAMTS5, respectively (*p* < 0.05). PC treatment also resulted in 44 % reduction in the immunostaining score of MMP-13 (*p* < 0.05). Although PC-E treatment resulted in reduction in the immunostaining score of MMP-13 (16 %), the difference did not reach statistical significance. PC and PC-E treatment resulted in 35 % and 40 % reductions in the immunostaining score of CCL-5, respectively (*p* < 0.05). Although PC and PC-E treatments resulted in reductions in the immunostaining score of Cox-2 (about 10-14 % reductions), the differences did not reach statistical significance.

### The effect of PC-E on gene expressions

We have shown that PC downregulated the expression of numerous genes classified in cell proliferation, angiogenesis, and inflammatory response while upregulating the expression of many genes classified in skeletal system development in the absence of calcium crystals [29]. To examine the molecular mechanisms underlying the decreased disease-modifying activity of PC-E, we compared the effect of PC-E on gene expressions with the effect of PC on gene expressions [29]. As shown in Table 1, PC-E downregulated almost all of the PC-downregulated genes classified in cell proliferation, suggesting that PC-E may inhibit cell proliferation as effectively as PC. However, PC-E had little effect on the expression of most of the PC downregulated genes classified in angiogenesis and inflammatory response, including prostaglandin-endoperoxide synthase 2 (PTGS2/Cox-2). PC-E also had little effect on the expressions of most of the PC upregulated genes classified in muscle tissue and skeletal system development (Table 2).

**Table 1:** Differentially expressed genes in PC-treated and PC-E-treated cells compared with untreated cells

Biological process	Gene name	Gene ID	Differ Expre (fold)* PC	Differ Expre (fold)** PC-E	Description
Cell proliferation					
	BLM	NM_000057	−3.64	−1.78	Bloom syndrome
	CCNE2	AF112857	−3.74	0.00	Cyclin E2
	CCNE1	AI671049	−2.30	0.00	Cyclin E1
	CDC25A	AY137580	−3.63	0.00	Cell division cycle 25 homolog A (S. pombe)
	CDC25C	NM_001790	−2.31	0.00	Cell division cycle 25 homolog C (S. pombe)
	CDC2	AA749427	−3.13	−3.42	Cell division cycle 2, G1 to S and G2 to M
	CDC6	NM_001254	−2.36	−9.19	Cell division cycle 6 homolog (S. cerevisiae)
	CDC7	NM_003503	−2.12	−4.42	Cell division cycle 7 homolog (S. cerevisiae)
	CDCA3	NM_031299	−1.83	−2.61	Cell division cycle associated 3
	CDCA5	BE614410	−2.41	−3.37	Cell division cycle associated 5
	CDCA7	AY029179	−2.36	−4.60	Cell division cycle associated 7
	CDCA8	BC001651	−2.11	−2.12	Cell division cycle associated 8
	CDK2	AB012305	−2.74	−3.36	Cyclin-dependent kinase 2
	NCAPH	D38553	−2.64	−2.21	Non-SMC condensin I complex, subunit H
	HELLS	NM_018063	−2.49	−4.77	Helicase, lymphoid-specific
	AURKB	AB011446	−2.43	0.00	Aurora kinase B
	KIF23	AW192521	−2.41	−3.83	Kinesin family member 23
	CLASP2	BC029035	−2.40	0.00	Cytoplasmic linker associated protein 2
	NUF2	AF326731	−2.35	−4.04	NUF2, NDC80 kinetochore complex component, homolog
	DSN1	NM_024918	−2.35	−3.09	DSN1, MIND kinetochore complex component, homolog
	SPC24	AI469788	−2.32	0.00	SPC24, NDC80 kinetochore complex component, homolog
	SPC25	AF225416	−2.10	−3.40	SPC25, NDC80 kinetochore complex component, homolog
	HMGA2	AI990940	−2.30	−3.41	High mobility group AT-hook 2
	LIG1	NM_000234	−2.25	−2.20	Ligase I, DNA, ATP-dependent
	KIFC1	BC000712	−2.21	−2.08	Kinesin family member C1
	BRCA2	X95152	−2.18	0.00	Breast cancer 2, early onset
	ERCC6L	NM_017669	−2.17	−2.29	Exc repair cross-comp repair deficiency, comp group 6-like
	SPAG5	NM_006461	−2.16	−2.61	Sperm associated antigen 5
	NEK2	Z25425	−2.14	−2.20	NIMA (never in mitosis gene a)-related kinase 2
	NCAPG	NM_022346	−2.12	−4.06	Non-SMC condensin I complex, subunit G
	ZWINT	NM_007057	−2.01	−3.93	ZW10 interactor antisense
	PARD3B	AF428251	3.24	1.68	Par-3 partitioning defective 3 homolog B (C. elegans)
	11-Sep	AI333326	2.28	0.00	Septin 11
Angiogenesis					
	NRP1	AF280547	−2.69	0.00	Neuropilin 1
	TEK	BF594294	−2.58	−1.73	TEK tyrosine kinase, endothelial
	ELK3	NM_005230	−2.42	0.00	ELK3, ETS-domain protein (SRF accessory protein 2)
	EREG	NM_001432	−1.90	0.00	Epiregulin
	PML	AW291023	−1.89	0.00	Promyelocytic leukemia
	COL15A1	NM_001855	−1.80	0.00	Collagen, type XV, alpha 1
	NRP2	AI819729	−1.75	0.00	Neuropilin 2
	SPHK1	NM_021972	−1.72	0.00	Sphingosine kinase 1
	FOXC2	NM_005251	−1.68	0.00	Forkhead box C2 (MFH-1, mesenchyme forkhead 1)
	SCG2	NM_003469	−1.66	2.54	Secretogranin II (chromogranin C)
	EDNRA	NM_001957	−1.56	0.00	Endothelin receptor type A
	TGFBR2	NM_003242	−1.51	−1.97	Transforming growth factor, beta receptor II (70/80 kDa)
	ROBO4	AA156022	−1.51	0.00	Roundabout homolog 4, magic roundabout (Drosophila)
	JAG1	AI457817	2.42	1.76	Jagged 1 (Alagille syndrome)
	NOTCH4	AI341271	1.75	0.00	Notch homolog 4 (Drosophila)
	RUNX1	D89788	1.73	0.00	Runt-related transcription factor 1
	EPAS1	NM_001430	1.67	0.00	Endothelial PAS domain protein 1
Inflammatory response					
	PTGS2	AY151286	−6.09	0.00	Prostaglandin-endoperoxide synthase 2
	SERPINA1	AF119873	−2.15	0.00	Serpin peptidase inhibitor, clade A
	GPR68	AI805006	−2.15	−2.44	G protein-coupled receptor 68
	BMPR1B	AA935461	−2.12	0.00	Bone morphogenetic protein receptor, type IB
	EVI1	BE466525	−2.00	−1.69	Ecotropic viral integration site 1
	FOS	BC004490	−1.92	1.51	V-fos FBJ murine osteosarcoma viral oncogene homolog
	IRAK2	AI246590	−1.82	0.00	Interleukin-1 receptor-associated kinase 2
	CCL2	S69738	−1.82	0.00	Chemokine (C-C motif) ligand 2
	CCR1	NM_001295	−1.60	0.00	Chemokine (C-C motif) receptor 1
	CXCL2	M57731	−1.66	0.00	Chemokine (C-X-C motif) ligand 2
	SPN	BC035510	−1.79	0.00	Sialophorin (leukosialin, CD43)
	TLR4	AF177765	−1.70	0.00	toll-like receptor 4
	SCG2	NM_003469	−1.66	2.54	Secretogranin II (chromogranin C)
	FN1	AJ276395	−1.58	−1.52	Fibronectin 1
	KLKB1	BE326857	−1.52	0.00	Cytochrome P450, family 4, subfamily V, polypeptide 2
	NDST1	NM_001543	2.05	0.00	N-deacetylase/N-sulfotransferase (heparan glucosaminyl) 1
	C3	NM_000064	2.05	0.00	Complement component 3
	SERPINA3	NM_001085	1.88	0.00	Serpin peptidase inhibitor, clade A
	SBNO2	AC005390	1.78	0.00	strawberry notch homolog 2 (Drosophila)
	NFKBIZ	BE646573	1.74	0.00	NF-KB inhibitor zeta
	MASP1	NM_001879	1.64	0.00	Mannan-binding lectin serine peptidase 1
	STAT5B	NM_012448	1.59	0.00	Signal transducer and activator of transcription 5B

*Negative number indicates decreased expression (fold) in treated hTERT-OA 13A FLS compared with untreated cells

Positive number indicates elevated expression (fold) in treated hTERT-OA 13A FLS compared with the untreated cells

**Dada was published previously [29]

**Table 2:** Differentially expressed genes in PC-treated and PC-E-treated cells compared with untreated cells

Biological process	Gene name	Gene ID	Differ Expre (fold)* PC	Differ Expre (fold)** PC-E	Description
Muscle tissue development					
	IGFBP5	AW157548	8.57	1.55	Insulin-like growth factor binding protein 5
	CACNB4	NM_000726	2.73	0.00	Calcium channel, voltage-dependent, beta 4 subunit
	TPM1	AI521618	2.43	0.00	Tropomyosin 1 (alpha)
	JAG1	U61276	2.02	0.00	Jagged 1 (Alagille syndrome)
	MORF4L2	H43976	1.90	0.00	Mortality factor 4 like 2
	NRG1	NM_013957	1.88	0.00	Neuregulin 1
	SIRT2	BG722779	1.86	0.00	Sirtuin (silent mating type information regulation 2 homolog) 2
	NF1	D12625	1.80	0.00	Neurofibromin 1
	OBSL1	BF446688	1.78	0.00	Obscurin-like 1
	MBNL1	AA732240	1.73	0.00	Muscleblind-like (Drosophila)
	TPM1	NM_000366	1.72	0.00	Tropomyosin 1 (alpha)
	CAV2	AA150110	1.67	0.00	Caveolin 2
	RXRA	BE675800	1.66	0.00	Retinoid X receptor, alpha
	NR2F2	AL554245	1.63	0.00	Nuclear receptor subfamily 2, group F, member 2
	TCF7L2	AV721430	1.61	2.00	Transcription factor 7-like 2 (T-cell specific, HMG-box)
	TBX2	U28049	−4.17	0.00	T-box 2
	ADRB2	NM_000024	−2.36	0.00	Adrenergic, beta-2-, receptor, surface
	SORT1	BE742268	−1.93	0.00	Sortilin 1
	GJC1	NM_005497	−1.77	−3.09	Gap junction protein, gamma 1, 45 kDa
	CENPF	U30872	−1.77	0.00	Centromere protein F, 350/400 ka (mitosin)
	BCL2	NM_000657	−1.71	1.55	B-cell CLL/lymphoma 2
	TBX3	U69556	−1.71	1.77	T-box 3
	SDC1	NM_002997	−1.65	−1.89	Syndecan 1
	TBX5	AW269421	−1.54	0.00	T-box 5
	RARB	NM_015854	−1.51	0.00	Retinoic acid receptor, beta
Skeletal development					
	ANXA2	D28364	2.17	0.00	Annexin A2
	VDR	AA772285	2.11	0.00	Vitamin D (1,25- dihydroxyvitamin D3) receptor
	GNAS	AI693143	1.95	1.60	GNAS complex locus
	ACAN	NM_001135	1.80	−1.51	Aggrecan
	COL1A1	AI743621	1.66	0.00	Collagen, type I, alpha 1
	COL1A2	AA628535	1.88	0.00	Collagen, type I, alpha 2
	COL11A1	NM_001854	1.50	0.00	Collagen, type XI, alpha 1
	COL12A1	AU146651	1.93	1.51	Collagen, type XII, alpha 1
	MSX2	D89377	1.85	0.00	Msh homeobox 2
	GHR	NM_000163	1.76	0.00	Growth hormone receptor
	MEF2C	AL536517	1.59	0.00	Myocyte enhancer factor 2C
	THRA	NM_003250	1.57	1.60	Thyroid hormone receptor, alpha
	RUNX2	AW469546	1.55	1.89	Runt-related transcription factor 2
	CLEC3B	NM_003278	1.55	3.26	Exosome component 7
	MEF2C	N22468	1.55	0.00	Myocyte enhancer factor 2C
	IGFBP4	NM_001552	1.54	0.00	Insulin-like growth factor binding protein 4
	PRKRA	AA279462	1.53	0.00	Protein kinase, interferon-inducible RNA dependent activator
	TNFRSF11B	NM_002546	1.50	−2.76	Tumor necrosis factor receptor superfamily, member 11b
	BMPR1B	AA935461	−2.12	0.00	Bone morphogenetic protein receptor, type IB
	ANKH	AF274753	−1.93	−1.71	Ankylosis, progressive homolog (mouse)
	ACVR2A	NM_001616	−1.89	0.00	Activin A receptor, type IIA
	CYTL1	NM_018659	−1.83	0.00	Cytokine-like 1
	TBX3	U69556	−1.71	1.77	T-box 3 (ulnar mammary syndrome)
	SOX9	NM_000346	−1.71	0.00	SRY (sex determining region Y)-box 9
	FOXC2	NM_005251	−1.68	0.00	Forkhead box C2 (MFH-1, mesenchyme forkhead 1)
	KIAA1217	BC017424	−1.66	0.00	KIAA1217
	MMP9	NM_004994	−1.61	0.00	Matrix metallopeptidase 9
	TGFBR2	NM_003242	−1.51	−1.97	Transforming growth factor, beta receptor II (70/80 kDa)

*Negative number indicates decreased expression (fold) in treated hTERT-OA 13A FLS compared with untreated cells

Positive number indicates elevated expression (fold) in treated hTERT-OA 13A FLS compared with the untreated cells

**Data was published previously [29]

## Discussion

PC, PC-E, and EHDP are powerful calcification inhibitors, suggesting that the phosphate group within these molecules play a key role in their calcification-inhibitory activity. These findings cast some doubt about the role of the calcification-inhibitory activity of these molecules in their OA disease-modifying activity because bisphosphonates displayed little disease-modifying effect on the animal model of OA [27, 28]. PC, consistent with previous findings [18, 26], is more powerful than EHDP as a calcification inhibitor, suggesting that the three carboxyl groups also play a key role in the calcification-inhibitory activity of PC. However, replacement of the β-carboxyl group with a β-ester group only resulted in a moderate 19 % reduction in the calcification-inhibitory activity of PC, indicating that three carboxyl groups are required for the strong calcification-inhibitory activity and that a single carboxyl group only plays a moderate role in the calcification-inhibitory activity of PC.

PC-E, similar to PC, inhibited the proliferation of OA FLSs. Consistent with this finding, PC-E downregulated the expressions of almost all of the PC-downregulated genes classified in cell proliferation as effectively as PC (Table 1). These findings indicate that the β-carboxyl group plays little role in the proliferation-inhibitory activity of PC and that the phosphate group within these molecules plays a key role in the proliferation-inhibitory activity [29]. Because PC-E is much less powerful than PC as an OA disease-modifying drug, it suggests that the proliferation-inhibitory activity of PC plays little role in its OA disease-modifying activity and that those PC downregulated genes classified in cell proliferation are unlikely key OA disease candidate genes.

Medial meniscal calcification is absent in the meniscectomied right knee and severe articular cartilage calcification is not observed in the Hartley guinea pigs [24]; therefore, OA in the meniscectomied right knee was mainly induced by meniscal injury and joint instability, and had less to do with pathological calcification. In this model of posttraumatic OA, PC significantly reduced cartilage damage and proteoglycan loss, demonstrating that PC is a disease-modifying drug for posttraumatic OA therapy. We should point out that our finding and conclusion contradict with a previous study. Cheung et al. found that PC had no significant effect on cartilage degeneration in partial meniscectomied rabbits, and concluded that PC is potentially a disease-modifying drug for calcification-induced OA, but not for non-calcification-induced OA [24]. One possible explanation for these contradicting findings is that different doses of PC were used in the two studies. A single weekly injection (40 mg/kg) was used in the previous study whereas two weekly injections of PC (40 mg/kg) were used in this study. It is worth noting that PC treatment did reduce the histological score of the medial tibia in the rabbit (from 9.8 ± 1.7 to 8.1 ± 2.2) [24]. If higher doses or more injections of PC per week was used, statistically significant difference might have been observed.

The molecular mechanisms underlying the disease-modifying effect of PC remain poorly understood. If PC exerted its disease-modifying effect solely by inhibiting the formation of articular crystals, PC should have displayed little disease-modifying effect on posttraumatic OA. Moreover, PC-E should have displayed only moderately decreased disease-modifying effect on posttraumatic OA compared to PC because PC-E is still a powerful calcification inhibitor. However, we found that PC significantly inhibited cartilage degeneration and that PC-E was 46 % less powerful than PC in the inhibition of cartilage degeneration in the meniscectomied right knee. These findings indicate that crystal-dependent action is unlikely the sole action underlying the disease-modifying effect of PC or PC-E. PC and PC-E likely exert their disease-modifying activity through both a crystal-dependent action and a crystal-independent action. Consistent with this mechanism, PC-E had no effect on the expressions of PC downregulated genes classified in angiogenesis and inflammatory response and PC upregulated genes classified in skeletal system development; therefore, resulting in decreased OA disease-modifying activity. Taken together, it suggests that the gene expression-modulatory activity of PC may play an important role in its OA disease-modifying activity.

We demonstrated that PC reduced the levels of ADAMTS5 and MMP-13 proteins. It is worth noting that the level of MMP-13 protein is much higher in the middle zone than in the superficial and calcified zones (Fig. 7). In human OA articular cartilage, calcium crystals are detected in the superficial and calcified zones [39–41]. If calcium crystals are also present in the superficial or calcified zones of articular cartilage in the guinea pigs and those crystals played a key role in the induction of MMP-13 expression [42], high level of MMP-13 protein should have been observed in the superficial zone or calcified zone. Similarly, if the reduction in the level of MMP-13 protein in PC treated guinea pigs was due to the inhibition of PC on the interaction between calcium crystals and chondrocytes [23], significant reduction in the level of MMP-13 protein should have been observed in the superficial zone or calcified zone, but not in the middle zone. However, these were not what we observed, indicating that calcium-containing crystals is unlikely a key inducer for the production of MMP-13 in the articular cartilage and that PC exerts its inhibitory effect on the production of MMP-13 through a crystal-independent action.

Our findings do suggest that crystal-dependent action of PC plays a role. For example, PC treatment resulted in a 46 % reduction in the histological scores of cartilage in meniscectomied right knee but a 54 % reduction in non-operated left knee. One explanation for this difference (46 % reduction via 54 % reduction) is that pathological meniscal calcification plays a role in the non-operated left knee OA [7, 43]. It is likely that PC exerts its disease-modifying effect on the posttraumatic OA in the right knee through a crystal-independent action whereas PC exerts its disease-modifying effect on the primary OA in the left knee through both a crystal-dependent action and a crystal-independent action. It is conceivable that inhibition of cartilage degeneration through a single action (inhibiting non-crystal-dependent disease pathway) is less effective than through 2 actions (inhibiting both crystal-dependent disease pathway and non-crystal-dependent disease pathway).

PC-E was 46 % less powerful than PC in the inhibition of cartilage degeneration in the non-operated left knee but was 33 % less powerful than PC in the inhibition of cartilage degeneration in the meniscectomied right knee. Again, this difference (46 % reduction via 33 % reduction) can be explained similarly. The replacement of β-carboxyl group with an ester group resulted in partial impairment of both crystal-dependent action and crystal-independent action of PC. It is conceivable that partial impairment of both actions would result in a greater loss in the disease-modifying effect on the primary OA in non-operated left knee than on the posttraumatic OA in meniscectomied right knee because crystal-dependent disease pathway was absent in the right knee.

Our study has limitations. One limitation is that the OA in the meniscectomied right knee is not absolutely a non-calcification-induced OA because crystals must be present in the calcified zone. However, the increased severity of cartilage damage in the meniscectomied right knee compared to the non-operated left knee indicate that the most severe cartilage lesions in the right knee medial tibia plateau, especially the cartilage lesions in the peripheral area, were caused by meniscal injury and joint instability and has little to do with pathological calcification. In addition, a previous study found that PC had no significant effect on crystal formation in cartilage [16]. Taken together, it indicates that PC inhibits cartilage degeneration, at least in part, through a crystal-independent action.

## Conclusions

Posttraumatic OA in Hartley guinea pigs are characterized by breakdown of collagen fibers and proteoglycan loss. PC is not only potentially a disease-modifying drug for calcification-induced OA therapy but also potentially a disease-modifying drug for posttraumatic OA therapy. PC exerts its disease-modifying activity on OA through two independent actions, a crystal-dependent action and a crystal-independent action. The β-carboxyl group plays little role in the proliferation-inhibitory activity and the modulatory effect on the expressions of genes classified in cell proliferation, but a major role in the OA disease-modifying activity and the modulatory effect on the expressions of genes classified in angiogenesis, inflammatory response, and skeletal system development of PC. The β-carboxyl group is not a group that should be used to link other active group(s) to create new PC analogues as OA disease-modifying drugs.

## Abbreviations

ADAMTS5: ADAM metallopeptidase with thrombospondin type 1 motif 5
CCL-5: chemokine (C-C motif) ligand 5
Cox-2: cyclooxygenase-2
EHDP: disodium ethane-1-hydroxy-1, 1-diphosphonate
FLSs: fibroblast-like synoviocytes
MMP-13: matrix metalloproteinase-13
OA: osteoarthritis
PC: phosphocitrate
PC-E: phosphocitrate-β-ethyl ester
SD: standard deviation
